# Changes in urinary output due to concomitant administration of sacubitril/valsartan and atrial natriuretic peptide in patients with heart failure: a multicenter retrospective cohort study

**DOI:** 10.1186/s40780-024-00379-1

**Published:** 2024-09-16

**Authors:** Tatsuki Yanagawa, Yuki Asai, Nobuyuki Zakoji, Shingo Hosoe, Yoshihiro Kondo, Shinnosuke Ootsuki, Hidekazu Kato, Maria Aoki, Yoshiaki Yamamoto, Takanori Yamamoto, Masaaki Takahashi

**Affiliations:** 1 National Hospital Organization Mie Chuo Medical Center, Tsu, Mie 514-1101 Japan; 2 Department of Pharmacy, Mie University Hospital, Faculty of Medicine, Mie University, 2-174 Edobashi, Tsu, Mie 514-8507 Japan; 3 National Hospital Organization Shizuoka Medical Center, 762-1 Nagasawa, Shimizu, Shizuoka, Sunto-gun 411-8611 Japan; 4 National Hospital Organization Toyohashi Medical Center, 50 Hamamichigami, Imure-cho, Toyohashi, Aichi 440-8510 Japan; 5 National Hospital Organization Nagoya Medical Center, 4-1-1 Sannomaru, Naka-Ku, Nagoya, Aichi 460-0001 Japan; 6 National Hospital Organization Kanazawa Medical Center, 1-1 Shimoishibiki, Kanazawa, Ishikawa 920-8650 Japan; 7 National Hospital Organization Nagara Medical Center, 1300-7 Nagara, Gifu, 502-8558 Japan; 8 National Center for Geriatrics and Gerontology, 7-430 Morioka, Obu, Aichi 474-8511 Japan; 9 Department of Clinical Research, National Hospital Organization Shizuoka Institute of Epilepsy and Neurological Disorders, 886 Urushiyama, Aoi-ku, Shizuoka, 420-8688 Japan; 10 National Hospital Organization Mie National Hospital, 357 Osatokubota, Tsu, Mie 514-0125 Japan

**Keywords:** Sacubitril/valsartan, Natriuretic peptide, Heart failure, Multicenter retrospective cohort study

## Abstract

**Background:**

Sacubitril/valsartan is an angiotensin receptor neprilysin inhibitor (ARNI) that inhibits the degradation of endogenous natriuretic peptides. Therefore, ARNIs may increase the efficacy of human atrial natriuretic peptide (hANP), a drug for acute heart failure, by mediating its pharmacological mechanism. This study was aimed at evaluating the effects of ARNIs on the pharmacological effects of hANP by using surrogate marker, such as urinary output, in patients with heart failure.

**Methods:**

In this multicenter retrospective cohort study, adult patients with heart failure who were taking angiotensin II receptor blockers (ARB) or ARNIs combined with hANP were enrolled. Information on basic characteristics, clinical laboratory data, medical history, and severity of cardiac insufficiency were collected from electronic medical records. The primary outcome was the change in adjusted fluid balance, calculated by IN-volume (mL/day) – OUT-volume (mL/day) / daily hANP dosage (μg).

**Results:**

Ninety-two and 62 patients in the ARB + hANP and ARNI + hANP groups, respectively, were eligible for analysis. The adjusted fluid balance in the ARNI + hANP group was significantly lower than that in the ARB + hANP group (*p* = 0.001). After propensity score matching, 27 patients from each group were included. Similarly, there was a significant reduction in adjusted fluid balance in the ARNI + hANP group after propensity score matching (*p* = 0.026).

**Conclusions:**

These findings suggest that ARNIs may enhance the efficacy of hANP and the combination of the two may be effective in the treatment of heart failure.

**Supplementary Information:**

The online version contains supplementary material available at 10.1186/s40780-024-00379-1.

## Background

Renin–angiotensin–aldosterone inhibitors, including angiotensin-converting enzyme inhibitors (ACEIs) and angiotensin II receptor blockers (ARBs), have been used for treating patients with heart failure (HF) with reduced ejection fraction (HFrEF) [1]. Furthermore, sacubitril/valsartan, an angiotensin receptor neprilysin inhibitor (ARNI), has been reported to reduce the risk of hospitalization and death associated with HF compared with those related to enalapril [2].Therefore, sacubitril/valsartan has been recommended for reducing morbidity and mortality in patients with HFrEF in the 2022 American Heart Association/American College of Cardiology/Heart Failure Society of America guideline [3].

Sacubitril/valsartan is decomposed into sacubitril and valsartan in the body [4]. While valsartan inhibits angiotensin II receptor-mediated vasoconstriction, leading to myocardial hypertrophy and fibrosis, and water and sodium reabsorption [5], sacubitril is further metabolized to the active neprilysin inhibitor. Neprilysin is a membrane-bound protease distributed in a wide range of tissues in the body and is responsible for the degradation of natriuretic peptides [6]. Atrial natriuretic peptides (ANPs) are known to contribute to a decrease in vascular tone, increase renally mediated excretion of electrolytes and water, and have antifibrotic and antihypertrophic effects in the heart [7].

In Japan, human ANPs (hANPs) have been administered intravenously to patients with acute HF to ensure urinary output [8]. Nougue et al. [9] reported that switching from an ACEI/ARB to an ARNI may increase blood ANP levels. Considering this evidence [9] and the pharmacological mechanism of ARNIs, neprilysin inhibition by sacubitril may elevate hANP concentrations in the body; however, the underlying mechanisms remain unknown.

Hence, this multicenter study was aimed at evaluating the effects of ARNIs on the pharmacological effects of hANPs based on the urinary output in patients with HF.

## Methods

### Study design

This multicenter, retrospective cohort study was conducted at seven hospitals belonging to the Tokai-Hokuriku Group of the National Hospital Organization (Mie Chuo Medical Center, Shizuoka Medical Center, Toyohashi Medical Center, Nagoya Medical Center, Kanazawa Medical Center, Nagara Medical Center, and National Center for Geriatrics and Gerontology).

### Data collection and exclusion criteria

Data on adult patients with HF receiving ARBs, ARNIs, and hANPs at the seven hospitals from September 1, 2020, to March 31, 2023, were collected. The exclusion criteria were as follows: (1) receipt of concomitant therapy for only 1 day, (2) unavailability of IN-volume (fluid intake and infusion volume) or OUT-volume (urinary output) measurements; (3) unknown ARB, ARNI, or hANP dosage; and (4) use of dialysis. Clinical data on patient characteristics (sex, age, weight, height, body surface area, clinical laboratory data, New York Heart Association [NYHA] classification, medical history, and concomitant medications); ARB, ARNI, and hANP dosages; ARB, ARNI, and hANP initiation dates; discharge and death dates; IN-volume; and OUT-volume were collected from electronic medical records. The data on concomitant medications were evaluated immediately prior to the initiation of combination therapy with ARB/ARNI and hANP. Adjusted fluid balance was calculated using the following formulae:

$$\text{Adjusted fluid balance}\ (\text{mL}/\text{day}/\upmu\text{g hANP}) = \text{IN} - \text{volume}\ (\text{mL}/\text{day})-\text{OUT}-\text{volume}\ (\text{mL}/\text{day})/\text{daily hANP dosage}(\upmu\text{g})$$

### Outcome

**Fig. 1:**
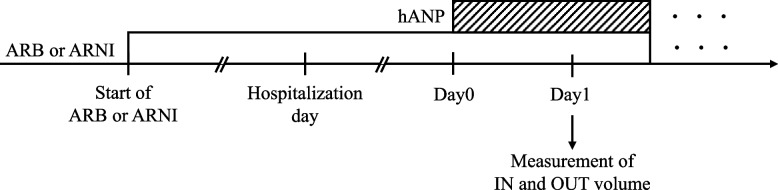
Schematic illustrating the study procedures. ARB, angiotensin II receptor blocker; ARNI, angiotensin receptor neprilysin inhibitor; hANP, human atrial natriuretic peptide; PS, propensity score

The design scheme used in this study is illustrated in Fig. 1. The start date of combination therapy with ARB/ARNI and hANP was defined as day 0. As the accurate time for conducting this combination therapy was unknown, and the diuretic may exert a maximal effect on day 1 [10], the fluid balance data were collected on day 1. The primary endpoint was the adjusted fluid balance. ARNIs increase endogenous ANP levels mediated by neprilysin inhibition, which leads to an increase in urinary output, without concomitant use of hANP [9, 11]. Whereas a diuretic effect of ARNIs was observed on the first day, this phenomenon was not observed on the fifth day [12]. Therefore, we evaluated the adjusted fluid balance in patients who received ARBs or ARNIs for more than 5 days, at the time of combination therapy with ARB/ARNI and hANP as sub-group analysis.

### Statistical analysis

As continuous variables were abnormally distributed, the Mann–Whitney *U* test was used. The chi-square test was used to compare categorical variables. To remove the influence of confounding factors, 1:1 propensity score (PS) matching was performed between the ARB + hANP and ARNI + hANP groups. The PS was calculated using the following variables: age; body mass index (BMI); creatinine clearance; medical history (atrial fibrillation, hypertension, diabetes, hyperlipidemia, and myocardial infarction); NYHA classification; and concomitant drugs such as loop diuretics, thiazide diuretics, tolvaptan, mineralocorticoid receptor antagonists (MRAs), sodium-glucose cotransporter 2 (SGLT2) inhibitors, and nitrates. The ARB + hANP and ARNI + hANP pairs were matched 1:1 with a caliper of 0.2 of the standard deviation of the logit of the PS. After PS matching, the respective baseline patient characteristics were evaluated and standardized differences were calculated. All statistical analyses were performed using SPSS version 28 (IBM Japan, Tokyo, Japan), and statistical significance was set at *p* < 0.05.

## Results

**Fig. 2:**
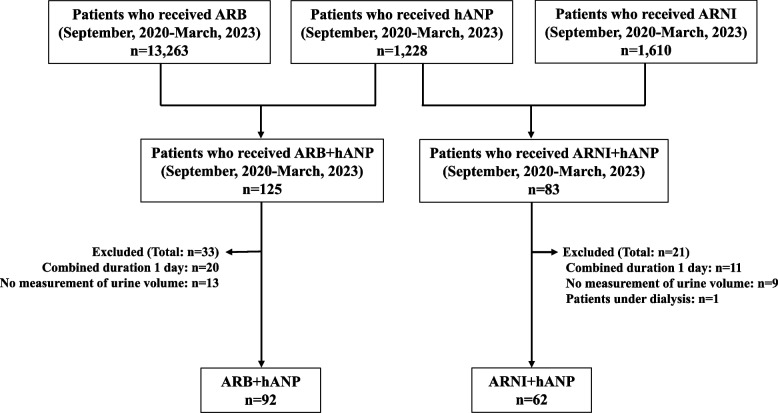
Flow diagram illustrating the patient recruitment process. ARB, angiotensin II receptor blockers; ARNI, angiotensin receptor neprilysin inhibitor; hANP, human atrial natriuretic peptide; PS, propensity score

From September 2020 to March 2023, 125 patients received ARBs and hANPs, and 83 patients received ARNIs and hANPs (Fig. 2). Based on the exclusion criteria, 92 and 62 patients in the ARB + hANP and ARNI + hANP groups, were eligible for the present study, respectively. The characteristics of patients in the ARB + hANP and ARNI + hANP groups are listed in Table 1. There were no significant differences in basic characteristics, such as sex, age, and clinical laboratory data, between the two groups. A higher percentage of patients in the ARB + hANP group had hypertension. A gap was observed between the NYHA classification (*p* < 0.001). Whereas the value of diastolic blood pressure was significantly lower in the ARB + hANP group compared to the ARNI + hANP group (*p* < 0.001), no significant differences were observed in systolic blood pressure. Regarding concomitant drugs, the ARNI + hANP group was more likely to use MRAs and SGLT2 inhibitors. The differences in tolvaptan and loop diuretics dosage between the ARB + hANP and ARNI + hANP groups were not significant.

**Table 1:** Baseline characteristics of the eligible patients

Factors	ARB + hANP	ARNI + hANP	*P* value
	*n* = 92	*n* = 62	
Basic property			
Sex (Male/Female)	56/36	41/21	0.507^a^
Age (years)	84 (77, 88)	82 (72, 88)^c^	0.263^b^
Body Weight (kg)	55.9 (48.2, 68.8)	57.3 (48.0, 68.5)^c^	0.897^b^
Height (m)	1.56 (1.47, 1.65)	1.61 (1.48, 1.67)^c^	0.110^b^
Body Mass Index (kg/m^2^)	23.2 (20.6, 26.6)	22.5 (19.4, 25.2)^c^	0.239^b^
Body surface area (m^2^)	1.54 (1.34, 1.72)	1.59 (1.40, 1.73)^c^	0.392^b^
Clinical laboratory data			
Na (mEq/L)	140 (136, 143)	140 (137, 143)^c^	0.601^b^
K (mEq/L)	4.3 (3.7, 4.7)	4.4 (3.8, 4.7)^c^	0.625^b^
Blood urea nitrogen (mg/dL)	26.7 (19.7, 38.0)	27.9 (20.0, 40.0)^c^	0.609^b^
Serum creatinine (mg/dL)	1.31 (0.93, 1.83)	1.39 (1.11, 1.79)^c^	0.202^b^
Creatinine clearance (mL/min)	30.5 (20.3, 46.1)	28.3 (22.1, 41.0)^c^	0.614^b^
Medical history			
Atrial fibrillation, n (%)	31 (34)	29 (47)	0.103^a^
Hypertension, n (%)	81 (88)	40 (65)	< 0.001^a^
Diabetes, n (%)	29 (32)	22 (35)	0.608^a^
Hyperlipidemia, n (%)	29 (32)	19 (31)	0.908^a^
Myocardial infarction, n (%)	14 (15)	12 (19)	0.501^a^
Valve replacement /Valvuloplasty, n (%)	5 (5)	2 (3)	0.519^a^
CABG, n (%)	5 (5)	1 (2)	0.402^a^
PCI, n (%)	10 (11)	7 (11)	0.935^a^
PMI, n (%)	5 (5)	5 (8)	0.516^a^
NYHA Classification			
Class I, n (%)	4 (4)	2 (3)	< 0.001^a^
Class II, n (%)	43 (47)	8 (13)	-
Class III, n (%)	31 (34)	37 (60)	-
Class IV, n (%)	6 (7)	9 (15)	-
Unknown, n (%)	8 (9)	6 (10)	-
Blood pressure			
Systolic blood pressure (mmHg)	120 (105, 133)	124 (108, 140)	0.051^b^
Diastolic blood pressure (mmHg)	62 (55, 72)	71 (65, 78)	< 0.001^b^
Oral medication			
Loop diuretic, n (%)	60 (65)	38 (61)	0.619^a^
Azosemide dosage (mg)	30 (30, 30)	30 (15, 30)	0.805^b^
Furosemide dosage (mg)	20 (20, 40)	20 (20, 40)	0.941^b^
Torasemide dosage (mg)	6 (4, 8)	8 (4, 8)	0.683^b^
Thiazide diuretic, n (%)	6 (7)	3 (5)	0.741^a^
Indapamide dosage (mg)	1 (0.75, 1)	1 (1, 1)	1.000^b^
Hydrochlorothiazide dosage (mg)	9.38 (7.81, 10.9)	-	-
Trichloromethiazide dosage (mg)	1 (1, 1)	1 (1, 1)	1.000^b^
Tolvaptan, n (%)	32 (35)	31 (50)	0.060^a^
Tolvaptan dosage (mg)	7.5 (3.75, 7.5)	7.5 (7.5, 15)	0.153^b^
MRA, n (%)	26 (28)	29 (47)	0.019^a^
Spironolactone dosage (mg)	25 (12.5, 25)	25 (25, 25)	0.435^b^
Eplerenone dosage (mg)	50 (50, 50)	50 (50, 50)	0.857^b^
Esaxerenone dosage (mg)	1.25 (1.25, 1.25)	1.25 (1.25, 1.25)	1.000^b^
β blocker, n (%)	37 (40)	31 (50)	0.231^a^
SGLT2 inhibitor, n (%)	10 (11)	22 (35)	< 0.001^a^
Empagliflozin dosage (mg)	10 (10, 10)	10 (10, 13.8)	0.494^b^
Dapagliflozin dosage (mg)	10 (10, 10)	10 (10, 10)	0.424^b^
Canagliflozin dosage (mg)	100 (100, 100)	100 (100, 100)	1.000^b^
Nitrate medicine, n (%)	4 (4)	4 (6)	0.715^a^
Number of internal medicine	8 (6, 11)	9 (7, 12)^c^	0.363^b^
Intravascular medication			
Catecholamine, n (%)	5 (5)	5 (8)	0.525^a^

*ARB* Angiotensin II receptor blocker, *ARNI* Angiotensin receptor neprilysin inhibitor, *CABG* Coronary artery bypass grafting, *hANP* human atrial natriuretic peptide, *MRA* Mineralocorticoid receptor antagonist, *NYHA* New York Heart Association, *PCI* Percutaneous coronary intervention, *PMI* Pacemaker implantation, *SGLT2* Sodium glucose cotransporter 2

^a^Chi–square test

^b^Mann–Whitney *U* test

^c^Each value represents the median (25th, 75th percentile)

**Fig. 3:**
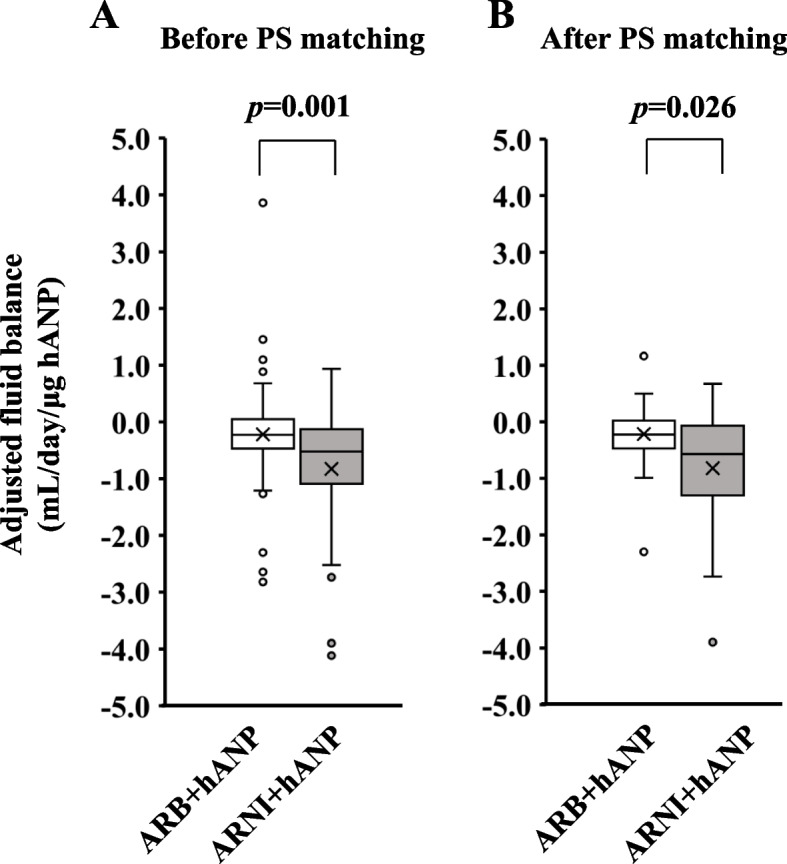
Fluid balance in the ARB + hANP and ARNI + hANP groups. **A** Adjusted fluid balance in the ARB + hANP and ARNI + hANP groups before PS matching. **B** Adjusted fluid balance in the ARB + hANP and ARNI + hANP groups after PS matching. Adjusted fluid balance was calculated as follows: In-volume (mL/day) – OUT-volume (mL/day) /daily hANP dosage. ARB, angiotensin II receptor blockers; ARNI, angiotensin receptor neprilysin inhibitor; hANP, human atrial natriuretic peptide; PS, propensity score

The adjusted fluid balance is shown in Fig. 3A. The adjusted fluid balance in the ARNI + hANP group was significantly lower than that in the ARB + hANP group (*p* = 0.001). In patients who received ARBs or ARNIs for more than 5 days, the adjusted fluid balance in the hANP + ARNI group also decreased on day 1 (*p* = 0.023) (Supplementary Fig. 1). In the sensitivity analysis, we compared the adjusted fluid balance after PS matching. After 1:1 PS matching, both ARB + hANP and ARNI + hANP groups included 27 patients each. Data on the basic characteristics of the patients after PS matching are shown in Table 2. There were no significant intergroup differences, and the standardized difference was less than 0.1, except for the BMI. A significant decrease in the adjusted fluid balance was observed in the ARNI + hANP group compared with that in the ARB + hANP group (*p* = 0.026) (Fig. 3B).

**Table 2:** Baseline characteristics after propensity score matching

Factors	ARB + hANP	ARNI + hANP	*P* value	Std diff
	n = 27	n = 27		
Basic property				
Age (years)	84 (78, 88)	84 (78, 90)^c^	0.749^b^	0.011
Body Mass Index (kg/m^2^)	23.5 (20.6, 269)	22.5 (20.0, 24.0)^c^	0.539^b^	0.120
Clinical laboratory data				
Creatinine clearance (mL/min)	30.1 (17.8, 41.0)	28.2 (23.2, 37.0)^c^	0.789^b^	0.049
Medical history				
Atrial fibrillation, n (%)	16 (59)	13 (48)	0.413^a^	0.043
Hypertension, n (%)	22 (81)	22 (81)	1.000^a^	0.000
Diabetes, n (%)	5 (19)	6 (22)	0.735^a^	0.021
Hyperlipidemia, n (%)	4 (15)	9 (33)	0.111^a^	0.086
Myocardial infarction, n (%)	5 (19)	5 (19)	1.000^a^	0.000
NYHA Classification				
Class I, n (%)	1 (4)	2 (7)	0.673^a^	0.036
Class II, n (%)	10 (37)	7 (26)	-	0.058
Class III, n (%)	14 (52)	14 (52)	-	0.000
Class IV, n (%)	2 (7)	4 (15)	-	0.052
Oral medication				
Loop diuretic, n (%)	21 (78)	20 (74)	0.750^a^	0.012
Thiazide diuretic, n (%)	1 (4)	2 (7)	1.000^a^	0.036
Tolvaptan, n (%)	14 (52)	14 (52)	1.000^a^	0.000
MRA, n (%)	12 (44)	11 (41)	0.783^a^	0.015
β blocker, n (%)	15 (56)	13 (48)	0.586^a^	0.029
SGLT2 blocker, n (%)	5 (19)	5 (19)	1.000^a^	0.000
Nitrate medicine, n (%)	1 (4)	1 (4)	1.000^a^	0.000

*ARB* Angiotensin II receptor blocker, *ARNI* Angiotensin receptor neprilysin inhibitor, *hANP* human atrial natriuretic peptide, *MRA* Mineralocorticoid receptor antagonist, *NYHA* New York Heart Association, *SGLT2* Sodium glucose cotransporter 2, *Std diff* Standardized difference

^a^Chi–square test.

^b^Mann–Whitney *U* test

^c^Each value represents the median (25th, 75th percentile)

## Discussion

To the best of our knowledge, this is the first study to demonstrate that concomitant use of ARNIs may increase urinary output compared to that of ARBs in patients treated with hANP. Moreover, the sensitivity analysis showed a decrease in the adjusted fluid balance, suggesting that the results of this study are highly reliable.

The ratio of hypertension, NYHA classification, diastolic blood pressure, MRA, and SGLT2 inhibitor were significantly different between the two groups (Table 1). The diuretic effect of MRA is relatively weak, and the beneficial effect of MRAs on HF has been attributed to non-diuretic effects [13]. On the other hand, SGLT2 inhibitors can increase urine output in HF patients [14]. Owing to these differences, we implemented PS matching to adjust for confounding factors, and differences in fluid levels were observed (Fig. 3B), suggesting that ARNI may potentially enhance the diuretic effect of hANP more effectively than ARB. In addition, this study included a large number of elderly patients (over the age of 80 years) compared with previous studies that investigated the background of patients with HF in Japan [15], indicating the efficacy of the ARNI and hANP combination in elderly patients with HF.

In sub-group analysis, we compared the adjusted fluid balance in patients who received ARBs or ARNIs for more than 5 days, at the time of combination therapy with ARB/ARNI and hANP to exclude the diuretic effect of ARNI alone. The adjusted fluid balance was lower in the ARNI + hANP group than in the ARB + hANP group, even after 5 days (Supplementary Fig. 1). Since hANP has an intramolecular neprilysin degradation site in its molecular structure [16, 17], it has been suggested that the suppression of hANP degradation by sacubitril results in an increased urinary output.

The present study has several limitations. First, the severity data of HF, such as clinical scenarios, serum ANPs, serum brain natriuretic peptides concentration, and ejection fraction data could not be elucidated because there are many missing values or descriptions. Second, although there were no significant differences in diuretic dosages among groups, the possibility that the combined effect of the diuretics influenced the fluid balance cannot be denied. Considering these limitations, future studies with larger sample sizes and greater statistical power should be conducted. Despite these limitations, the strength of this study was that it was a multicenter study, which minimized the scientific rigor and external validity biases.

## Conclusions

This study indicates that ARNIs may potentiate the action of hANPs and that the combination of ARNIs and hANPs may be useful in the treatment of HF.

## Supplementary Information

Supplementary Material 1: Fig. 1. Adjusted fluid balance in patients in whom the concomitant therapy was administered for more than 5 days. Adjusted fluid balance was calculated as follows: In-volume (mL/day) – OUT-volume (mL/day) /daily hANP dosage. ARB, angiotensin II receptor blockers; ARNI, angiotensin receptor neprilysin inhibitor; hANP, human atrial natriuretic peptide; PS, propensity score.

## Data Availability

The data supporting the findings of this study are available from the corresponding author upon reasonable request.

## Abbreviations

ACEI: Angiotensin-converting enzyme inhibitors
ANP: Atrial natriuretic peptide
ARB: Angiotensin II receptor blockers
ARNI: Angiotensin receptor neprilysin inhibitor
BMI: Body mass index
CABG: Coronary artery bypass grafting
hANP: Human atrial natriuretic peptide
HF: Heart failure
HFrEF: HF with reduced ejection fraction
MRA: Mineralocorticoid receptor antagonist
NYHA: New York Heart Association
PCI: Percutaneous coronary intervention
PMI: Pacemaker implantation
PS: Propensity score
SGLT2: Sodium-glucose cotransporter 2
