# Supplementary figures and images for: Changes in urinary output due to concomitant administration of sacubitril/valsartan and atrial natriuretic peptide in patients with heart failure: a multicenter retrospective cohort study
Source: J Pharm Health Care Sci. 2024 Sep 16;10:56. doi: 10.1186/s40780-024-00379-1 (PMC11403827; doi:10.1186/s40780-024-00379-1)

## Slide 1
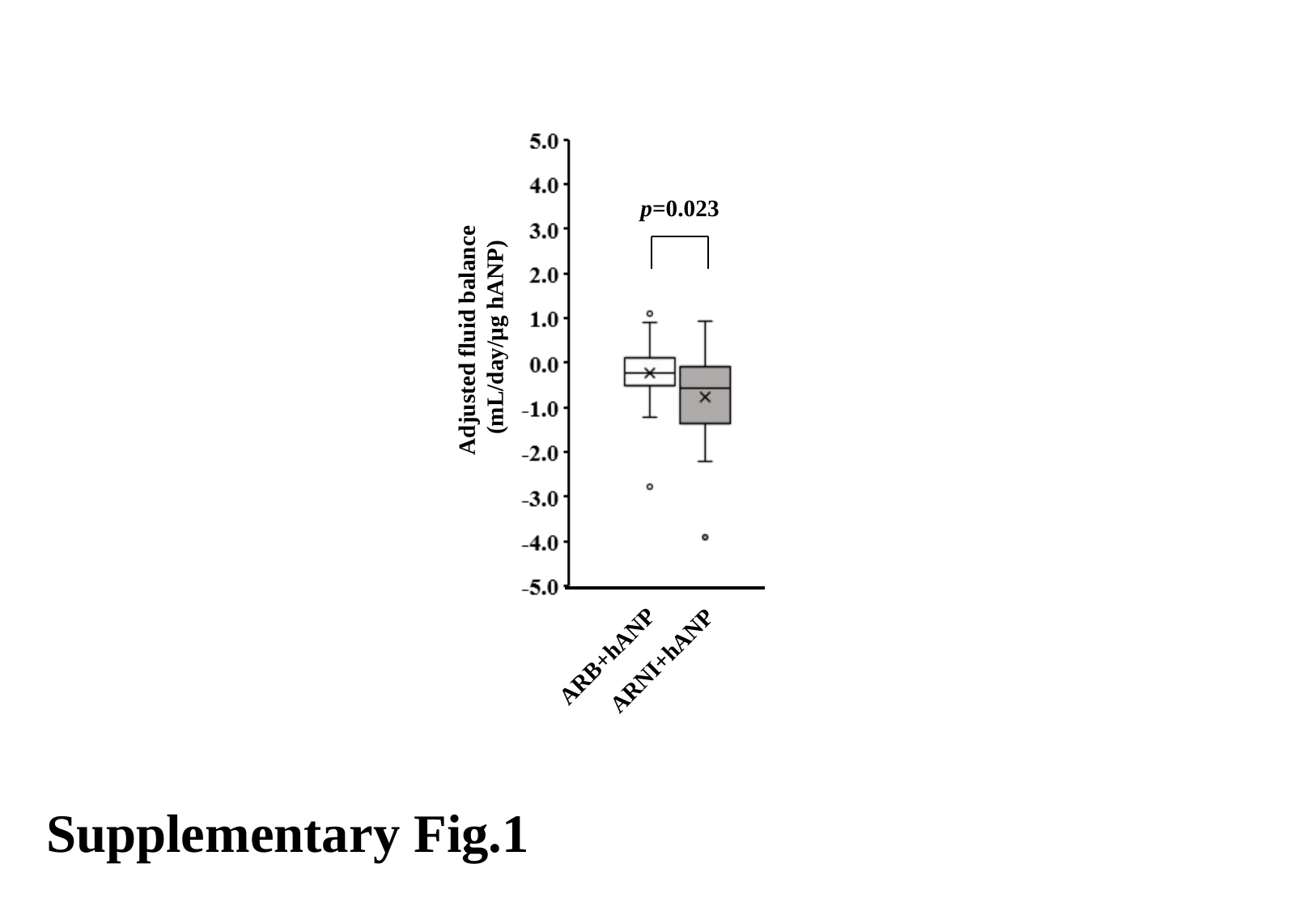

p=0.023
Adjusted fluid balance
 (mL/day/μg hANP)
ARB+hANP
ARNI+hANP
Supplementary Fig.1

Supplement: Supplementary file 1 — Supplementary Material 1: Fig. 1. Adjusted fluid balance in patients in whom the concomitant therapy was administered for more than 5 days. Adjusted fluid balance was calculated as follows: In-volume (mL/day) – OUT-volume (mL/day) /daily hANP dosage. ARB, angiotensin II receptor blockers; ARNI, angiotensin receptor neprilysin inhibitor; hANP, human atrial natriuretic peptide; PS, propensity score. [file 40780_2024_379_MOESM1_ESM.pptx]
